# If Veratrum Viride Is a Sure Antidote for Opium Poisoning in the Acute Form, May It Not Be in Chronic, Opium Inebriety?—an Affirmative Opinion

**Published:** 1880-06-20

**Authors:** J. S. Haldeman

**Affiliations:** Ohio


### IF VERATRUM VIRIDE IS A SURE ANTIDOTE FOR OPIUM POISONING IN THE ACUTE FORM, MAY IT NOT BE IN ITS CHRONIC, OPIUM INEBRIETY?—AN AFFIRMATIVE OPINION.

BY J. S. HALDEMAN, M.D., OF OHIO.

I received a communication, December 16, 1879, from J. B. Mattison, M.D., one of the managers and physicians of Parish Hall, Brooklyn, N. Y., a private medical home for opium habitues; and assistant physician "Sanitarium," Media, Pa., speaking praiseworthily of my articles on morphia poisoning, which appeared in the Cincinnati Lancet and Clinic; in which he asked, if I knew "anything regarding it in the chronic form; opium inebriety strictly speaking; and what is your idea as to the *modus operandi?* Are you inclined to think it would be very valuable in chronic opium taking?"

My reply to him was substantially as follows:

*Dear Doctor*, I will endeavor to answer your interrogatories to the best of my ability. First, you wish to know, if I have any knowledge of the virtues of the veratrum viride in the chronic form, or opium inebriety. I would answer I have not practically. I can only give you my opinion theoretically; which would be answering your second question, to wit: what is "my idea as to the *modus operandi?*"

The two articles, opium and veratrum, have evidently antipodal effects, as much so perhaps, as any other two in the materia medica. Therefore I should think that the veratrum would be the leading article in removing the pernicious effects of its antagonist, opium.

Opium is a direct stimulant and an indirect sedative of the nervous, muscular, and vascular system. On the other hand, veratrum viride excites the entire secretory system; hence it is emetic, expectorant, diaphoretic, and diuretic. It also stimulates the nerves, and hence is a nervine. It reduces and calms the circulation, and hence is an arterial sedative.

Now, when opium is administered for the first few hours, if the dose is not inordinately large, it augments the volume and velocity of the pulse; elevates the temperature; and increases the circulation, producing a general stimulation of the whole system. But after perhaps from twelve to twenty-four hours the giving of the drug, as a rule, sedation obtains, and the entire vital force of the nervous, muscular, and vascular system is lowered, and which calls imperiously for a repetition of it. As a sequence the secretions are measurably arrested through its contractile power over the muscular fibres. This, perhaps, accounts for the fact, that the opium user has always a more or less cadaverous appearance, showing plainly the diminution of the secretions, and in connection a change of the normal proportions of the globules of the blood.

Then the question would naturally arise in the mind of the physician, who wishes to remove this condition of things; what article as a remedy would he resort to? The question is plain, certainly the one that would bring a counter state of things; and that one is the veratrum viride, as I have already shown, though it may be faintly.

Dear Doctor, I think I have given you the *modus operandi*, which is what you asked me to do. I shall not essay to reason out the *modus operandi* in full detail, which would be an unpleasant task, and doubtless might prove unprofitable both to the writer and the parties addressed, for the reason that I have not the time at present, nor the space.

I do not know that the veratrum viride will beget a "loathing" for opium in the person who uses it, but I think it might cause an indifference for it, from the consideration that it will ultimately remove the necessity for its use—the peculiar condition in the system that opium establishes demanding its repetition. It may take the veratrum some time to accomplish this, therefore the remedy must be used delinquently —off and on—until recovery is fully brought about.

In regard to the length of time, for the continuance of the remedy, which was also inquired after, and which would be regarded by me, to depend very much upon circumstances. The idiosyncrasy, in different individuals, varies so constantly and greatly, that the susceptibility to the medicine, in one affected, might be greater than in another, consequently the time in the former would be shorter than in the latter in effecting a cure. If there is any bettering appreciable in the patient during the first two weeks or month treatment, it should be continued another two weeks or month, and so on, until a cure is obtained. But if there is no seeming or real improvement, during the first month or two, I think the treatment might as well be abandoned, with that particular patient, at any rate, for that time. The future, even in such a case, might develop altogether a different diathesis, so that a renewal of the effort would be justified and might be successful.

And now, as it relates to the size and frequency of the dose, and manner of its administration, whether hypodermically or by the mouth, which were also inquired after, I would say, in regard to them, we have to be governed greatly by circumstances, and the peculiar habitudes of the patients. There may be times when the patient might require minimum doses, and then again medium or maximum. Its effects must be closely watched; and be given in frequency and quantity, until it produces slight nausea; and then reduced and even suspended for a time, if need be. The dose might vary from one drop, and even less, to six or ten, or fifteen and more, if bearable, once, twice, or thrice daily.

All things being equal, I think it decidedly preferable to administer it by the mouth. In the acute form this cannot always be done. But in the chronic it would seldom be impracticable. There is not much danger, when watchfully administered, of any toxical results. During the treatment, the better plan is to withdraw the opium, little by little from the beginning, until it becomes safe to withold it altogether, or the indifference of which I spoke obtains. If need be, support the patient after the withdrawal of the opium by tonics, such as quinine, etc.

To me, there appears to be some ambiguity in the quotation you give from Dr. E. H. Sholl :

"In his case there came a gradual loathing of the opium, by which he was enabled rapidly to withdraw the drug—because the system would not tolerate it."

There came a loathing for opium, and in consequence he was enabled to withdraw the drug. What drug? The opium or veratrum? I may infer it means the latter. The system would not tolerate the depressing effects of it, after the stimulation produced by the opium had passed off.

If so, then tonics were indicated, and should have been given, to take the place of the abandoned stimulus to which it had been so long accustomed, and too in quantity in proportion to the amount of ennui occasioned.—*Cin. Lancet and Clinic.*

